# 超高效液相色谱-三重四极杆质谱法测定心脏组织中腺苷含量

**DOI:** 10.3724/SP.J.1123.2023.09016

**Published:** 2024-04-08

**Authors:** Zuoyin ZHU, Wenbo GUO, Hanke ZHAO, Jie WANG, Junhua YANG, Xinli ZHOU

**Affiliations:** 1.上海市农业科学院农产品质量标准与检测技术研究所, 上海 201403; 1. Institute for Agro-Food Standards and Testing Technology, Shanghai Academy of Agricultural Sciences, Shanghai 201403, China; 2.上海理工大学健康科学与工程学院, 上海 200093; 2. School of Health Science and Engineering, University of Shanghai for Science and Technology, Shanghai 200093, China

**Keywords:** 超高效液相色谱-三重四极杆质谱, 腺苷, 心脏组织, ultra performance liquid chromatography-triple quadrupole mass spectrometry (UPLC-MS/MS), adenosine (Ado), cardiac tissue

## Abstract

采用超高效液相色谱-三重四极杆质谱(UPLC-MS/MS)建立了一种灵敏度高、特异性好,且能够快速准确检测心脏组织中腺苷含量的方法。将样品溶于含10 μmol/L稳定剂2-羟基-3-壬基腺嘌呤盐酸盐(EHNA)的1 mL超纯水中,低温研磨2 min, 60 Hz冰水浴超声萃取40 min。以甲醇和5 mmol/L醋酸铵溶液为流动相,流速为0.4 mL/min,柱温为40 ℃,进样量为3 μL,采用电喷雾离子源(ESI)正离子切换多反应监测(MRM)模式对心脏组织中的腺苷进行定性定量分析,以标准曲线外标法准确定量。结果显示,在检测范围内线性关系拟合较优,线性范围为0.1~160 ng/mL,相关系数(*r*^2^)为0.9930,检出限(LOD)为0.03 ng/mL,定量限(LOQ)为0.1 ng/mL;腺苷在鼠心脏组织低、中、高3个水平的加标回收率分别为113.6%、96.3%、102.9%,日内精密度为1.7%~8.4%,日间精密度为2.6%~7.4%。相关性和一致性分析结果表明,UPLC-MS/MS法和双抗体夹心法测量结果存在正偏倚,两种方法腺苷检测结果呈极显著的正相关(*P*<0.0001);采用该方法随机测定17份小鼠和17份大鼠心脏样品,小鼠和大鼠心脏组织中腺苷的含量范围为3.25~8.78 mg/kg和10.24~15.19 mg/kg,腺苷的平均含量分别为5.37 mg/kg和12.60 mg/kg。本研究为心脏组织腺苷含量的测定提供了一种简单、精确、高效的检测方法,可为临床研究和疾病诊断提供重要的技术支持。

## Abstract (en)

A method based on ultra performance liquid chromatography-triple quadrupole mass spectrometry (UPLC-MS/MS) was developed and validated for the rapid and accurate determination of adenosine (Ado) in cardiac tissues with high sensitivity and specificity. The samples were dissolved in 1 mL of ultrapure water containing 10 μmol/L 2-hydroxy-3-nonyladenine hydrochloride (EHNA) as a stabilizer, ground at low temperature for 2 min, and then ultrasonically extracted at 60 Hz in an ice-water bath for 40 min. Methanol and 5 mmol/L ammonium acetate solution were used as the mobile phases under a flow rate of 0.4 mL/min, a column temperature of 40 ℃ and an injection volume of 3 μL. The Ado in cardiac tissue was qualitatively and quantitatively analyzed by electrospray ionization (ESI) positive-ion-switching in multiple reaction monitoring (MRM) mode. A solvent standard curve and the external standard method were used for the accurate quantification of Ado. The results showed that the matrix effect of Ado in cardiac tissue was very low. A good linear relationship was obtained in the range of 0.1-160 ng/mL, and the correlation coefficient (*r*^2^) was 0.9930. The limits of detection (LOD) and quantification (LOQ) were 0.03 and 0.1 ng/mL, respectively. The spiked recoveries of Ado in murine cardiac tissue were 113.6%, 96.3%, and 102.9% at three spiked levels of low, medium, and high, respectively. The intra-day repeatability (RSDs) were 1.7%-8.4%, and the inter-day reproducibility (RSDs) were 2.6%-7.4%. Based on the correlation and consistency results, a positive bias was observed between the proposed UPLC-MS/MS method and the double-antibody sandwich method. Moreover, the Ado contents detected by these two methods were significantly positively correlated (*P*<0.0001). Cardiac tissue samples were collected from 17 mice and 17 rats and detected in our laboratory. The content ranges of Ado in the cardiac tissues of mice and rats determined by the developed UPLC-MS/MS method were 3.25-8.78 mg/kg and 10.24-15.19 mg/kg, respectively (average adenosine contents: 5.37 and 12.60 mg/kg, respectively). The developed method is simple, accurate, sensitive, and it is suitable for the determination of Ado in cardiac tissues. It also provides important technical support for cardiac clinical research and disease diagnosis.

腺苷(adenosine, Ado)是核苷的一种,广泛分布于人与动植物体内。作为一磷酸腺苷(adenosine monophosphate, AMP)、二磷酸腺苷(adenosine diphosphate, ADP)、三磷酸腺苷(adenosine triphosphate, ATP)等化合物转化及合成的重要中间体,腺苷通过含量变化参与能量平衡^[1]^。在心血管系统、神经系统以及肌肉中,腺苷具有非常重要的生理功能,可以转化为环磷酸腺苷进行信号传递,参与调节物质代谢和生物学功能^[2]^。心肌内磷酸化后的腺苷不仅可以为心肌提供能量,还可以诱导冠状动脉血管扩张^[3]^。中枢神经系统中,腺苷可以作为神经调节介质,抵抗缺血性与疾病性神经伤害,也可以作为信号调节大脑能量的输入与输出^[4,5]^。正常生理状况下,细胞外的腺苷浓度恒定,基本维持在nmol/L范围,若细胞外腺苷水平过高,会被转运至细胞内,在细胞内腺苷转化为AMP或降解为肌苷。但在癫痫、缺血、疼痛、炎症和癌症等病理状态下,新陈代谢需求增加或者缺氧都会引起腺苷浓度升高^[6]^。此外,腺苷脱氢酶活性与许多疾病的诊断密切相关,其底物腺苷的浓度是反映该酶活性水平的重要标志物^[7]^。因此,临床上把腺苷浓度变化作为评估冠状动脉血流储备及微血管功能的重要指标,故血液和组织中腺苷含量的准确检测极为重要。

目前,传统的腺苷检测方法有薄层色谱法^[8]^、毛细管电泳法^[9,10]^、电化学法^[11]^、高效液相色谱法^[12,13]^等,但检测对象大多都集中在血液、尿液、虫草、保健食品等其他基质^[14⇓⇓⇓⇓-19]^,关于检测动物组织样品中腺苷含量的相关文献报道极少。与传统的检测方法(薄层色谱法、毛细管电泳法)相比,超高效液相色谱-三重四极杆质谱法(ultra performance liquid chromatography-triple quadrupole mass spectrometry, UPLC-MS/MS)操作更简单,响应快,灵敏度高,选择性强;与电化学适配体快速检测方法相比,UPLC-MS/MS对样品前处理要求不高,处理过程简单且不需要样品衍生化,方法建立成本低;与高效液相色谱法相比,UPLC-MS/MS通量更高,更高效,因此UPLC-MS/MS是检测腺苷的首选方法。

为改善目前腺苷检测技术的不足,填补组织样品中腺苷检测方法的空白,本文提出了一种简单且稳定的前处理方法,用于提取小鼠和大鼠心脏组织样品中的腺苷。通过UPLC-MS/MS,建立了一种灵敏度高、特异性好、操作简单,且能够快速准确检测组织样品中腺苷含量的方法。用于检测心脏组织中的腺苷浓度,为临床研究和疾病诊断提供技术支撑。

## 1 实验部分

### 1.1 仪器和试剂

Xevo-TQS超高效液相色谱-三重四极杆质谱仪(美国Waters公司); Wonbio-E多样品冷冻研磨仪(上海万柏公司); AL104分析天平(美国梅特勒-托利多仪器有限公司); Milli-Q超纯水仪(美国Millipore公司); SK8210LHC超声波清洗机(上海科导超声仪器有限公司); ST16R台式冷冻离心机(美国Thermo Fisher公司); Infinite M200Pro酶标仪(瑞士Tecan公司)。

乙腈、甲醇(纯度≥99%,德国Merck公司);腺苷标准品、醋酸铵(纯度≥99%)、高氯酸(分析纯)(美国Sigma-Aldrich公司); 2-羟基-3-壬基腺嘌呤盐酸盐(hydrochloride, EHNA,纯度≥98%,美国MedChemexpress公司);心脏组织样品:随机选取实验室SD大鼠和BALB/c小鼠的心脏样品,低温保存于-80 ℃。

本实验用鼠均得到上海市农业科学院实验动物伦理委员会批准,批准文号为SAASPZ0921034。

### 1.2 样品前处理

准确称取50.0 mg心脏组织样品,置入2 mL离心管中,加入1 mL超纯水和100 μL EHNA腺苷稳定剂(10 μmol/L),浸泡3 min后,涡旋1 min。在离心管中加入两粒小钢珠,低温研磨2 min, 60 Hz冰浴超声提取40 min,以12000 r/min离心3 min,吸取上清液,经0.22 μm滤膜过滤后,取200 μL滤液于2 mL离心管中,用超纯水稀释至1 mL,上机测定。空白心脏样品加标处理:选取经检测不含腺苷的空白心脏组织样品,称取50.0 mg于2 mL离心管中,加入110 μL标准溶液(1 μg/mL)后,按上述提取方法进行样品前处理。

### 1.3 腺苷标准溶液的配制

准确称取2.0 mg腺苷标准品,充分溶解于1 mL超纯水中,得到2 mg/mL的标准储备液,于-20 ℃保存。将标准储备液用空白心脏样品提取液和超纯水分别梯度稀释为160、80、40、20、10、1、0.1 ng/mL的系列标准工作溶液。

### 1.4 UPLC-MS/MS检测条件

使用Waters XBridge BEH C_18_柱(100 mm×3 mm, 2.5 μm);流动相A:甲醇,流动相B: 5 mmol/L醋酸铵水溶液。梯度洗脱程序:0~3 min, 5%A; 3~5 min, 30%A~90%A; 5~6 min, 90%A; 6~6.1 min, 90%A~5%A; 6.1~8 min, 5%A。流速0.4 mL/min;进样量3 μL;柱温40 ℃。采用电喷雾电离源(ESI),正离子模式扫描;雾化气、辅助气均为高纯氮气;碰撞气为高纯氩气;毛细管界面电压为2.5 kV;雾化温度为500.0 ℃;离子源温度为150 ℃;雾化气体压力为0.07 MPa;脱溶剂气体流速为1000 L/h。通过多反应监测(MRM)模式对心脏组织中腺苷进行定量分析。腺苷的保留时间、母离子、子离子、锥孔电压、碰撞能量等质谱参数见表1。数据分析采用UPLC-MS/MS配套使用的MassLynxv4.1和Targetlynx软件处理。

**表 1:** 腺苷的保留时间和质谱参数

Sample	t_R_/min	Precursor ion (m/z)	Product ions (m/z)	Cone voltage/V	Collision energies/V
Ado	3.01	268.129	119.098^*^/136.133	28	40/16

* Quantitative ion.

### 1.5 双抗体夹心法测定小鼠心脏腺苷含量

用北京某公司商用的双抗体夹心法测定鼠心脏组织中腺苷含量。使用纯化的鼠腺苷抗体涂覆微孔板,制备成固定的抗体层,在涂覆好的微孔中加入腺苷样本,与辣根过氧化物酶标记的腺苷抗体相互结合,构建抗体-抗原-酶标记抗体复合物,进行充分洗涤清除未结合的杂质成分后,添加3,3',5,5'-四甲基联苯胺底物进行显色。在辣根过氧化物酶的催化下,3,3',5,5'-四甲基联苯胺被转化成蓝色,并在酸性条件下进一步变成黄色。使用酶标仪,在450 nm波长下测定吸光度,测定样品中腺苷的含量。

### 1.6 一致性分析

应用GraphPad Prism 9.0.0软件中的Bland-Altman散点图和Pearson相关性,分析UPLC-MS/MS法和双抗体夹心法测量结果的一致性。Bland-Altman散点图:以两种方法测量结果的差值(*d*)为纵坐标,两种方法测量结果的均值为横坐标。*d*的变异情况用差值的标准差来描述,95%差值应该位于*d*±1.96之间,即95%为一致性界限(LOA)。当95%的点都在一致性区间内,则认为这两种方法具有较好的一致性。

## 2 结果与讨论

### 2.1 方法优化

#### 2.1.1 色谱条件的优化

考察了Waters XBridge BEH C_18_(100 mm×3 mm, 2.5 μm)、Waters Acquity UPLC BEH C_18_(100 mm×2.1 mm, 1.7 μm)两种不同型号、不同粒径的色谱柱的分离效果。结果表明,当流动相为5 mmol/L醋酸铵水溶液-甲醇时,Waters XBridge BEH C_18_色谱柱(图1a)对腺苷的分离效果较好,色谱峰更加对称尖锐,要明显优于Waters Acquity UPLC BEH C_18_色谱柱(图1b);当色谱柱为Waters XBridge BEH C_18_柱、流动相为5 mmol/L醋酸铵水溶液-甲醇时(图1a),分离效果优于流动相为0.1%甲酸水-甲醇时(图1c),腺苷的色谱峰形和响应强度最好,保留时间稳定;这可能是在流动相中加入5 mmol/L醋酸铵,使腺苷分子更好的离子化。分子的离子化程度越高,目标物的响应程度越高,峰形就越好^[20]^。

**图 1:**
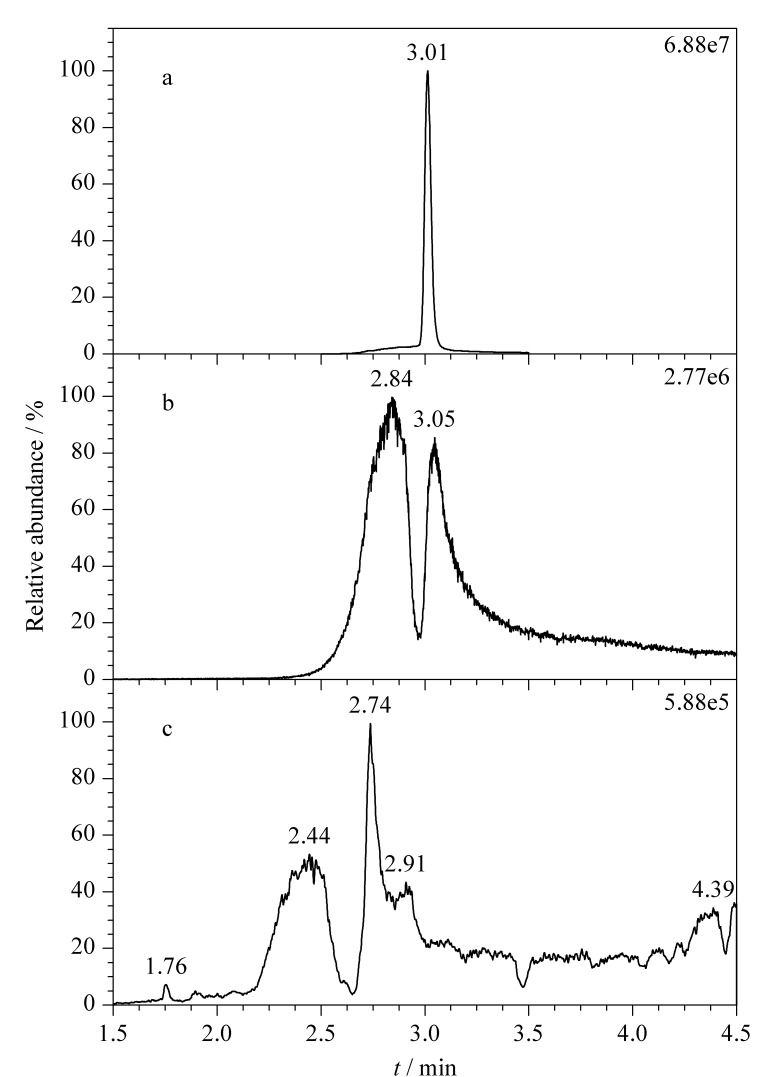
腺苷在不同色谱柱和流动相条件下的MRM色谱图

#### 2.1.2 质谱条件的优化

通过单进样方式优化质谱条件,在ESI^+^模式下扫描(*m/z* 100~800),选择母离子。在确定母离子的基础上,进行子离子扫描,通过比较响应值选择2个子离子碎片。利用母离子的信号强度优化锥孔电压、子离子的信号强度优化碰撞能量,最终得到的母离子、子离子、锥孔电压和碰撞能量如表1所示。在最佳质谱条件的基础上建立了MRM扫描模式,对心脏组织中的腺苷,以响应值高的通道作为定量离子通道,响应值略低的作为定性离子通道。

#### 2.1.3 提取剂的优化

由于体内组织中腺苷快速形成且快速清除,因此腺苷的半衰期很短^[21,22]^,需在提取剂中加入100 μL EHNA稳定剂(10 μmol/L)来预防腺苷代谢及其在提取过程中降解。为了更好地提取和检测组织中的腺苷,本文比较了5种提取剂对心脏组织中腺苷提取和检测效果的影响(图2a~e)。结果发现,超纯水为提取剂时,腺苷峰形最好,无杂峰,无拖尾现象,响应值高,为4.64×10^7^,基线噪声较小(图2a);提取剂为乙腈-水(20∶80, v/v,图2b)、乙腈-水(80∶20, v/v,图2c)和纯乙腈(图2d)时,Ado响应值较高,但是色谱峰拖尾严重,随着提取剂中乙腈含量的增加,腺苷出峰效果逐渐变差,出现杂峰;70%的高氯酸为提取剂时,响应值最低,为1.59×10^6^,保留时间偏移严重,同时出现较多杂峰,且基线噪声较大(图2e),这可能是高氯酸破坏了心脏组织中的蛋白质等生物大分子,大分子进一步分解,导致提取液中杂质较多,干扰腺苷出峰。综上,纯水对腺苷具有更好的提取效果,因此选择超纯水为提取剂进行后续实验。

**图 2:**
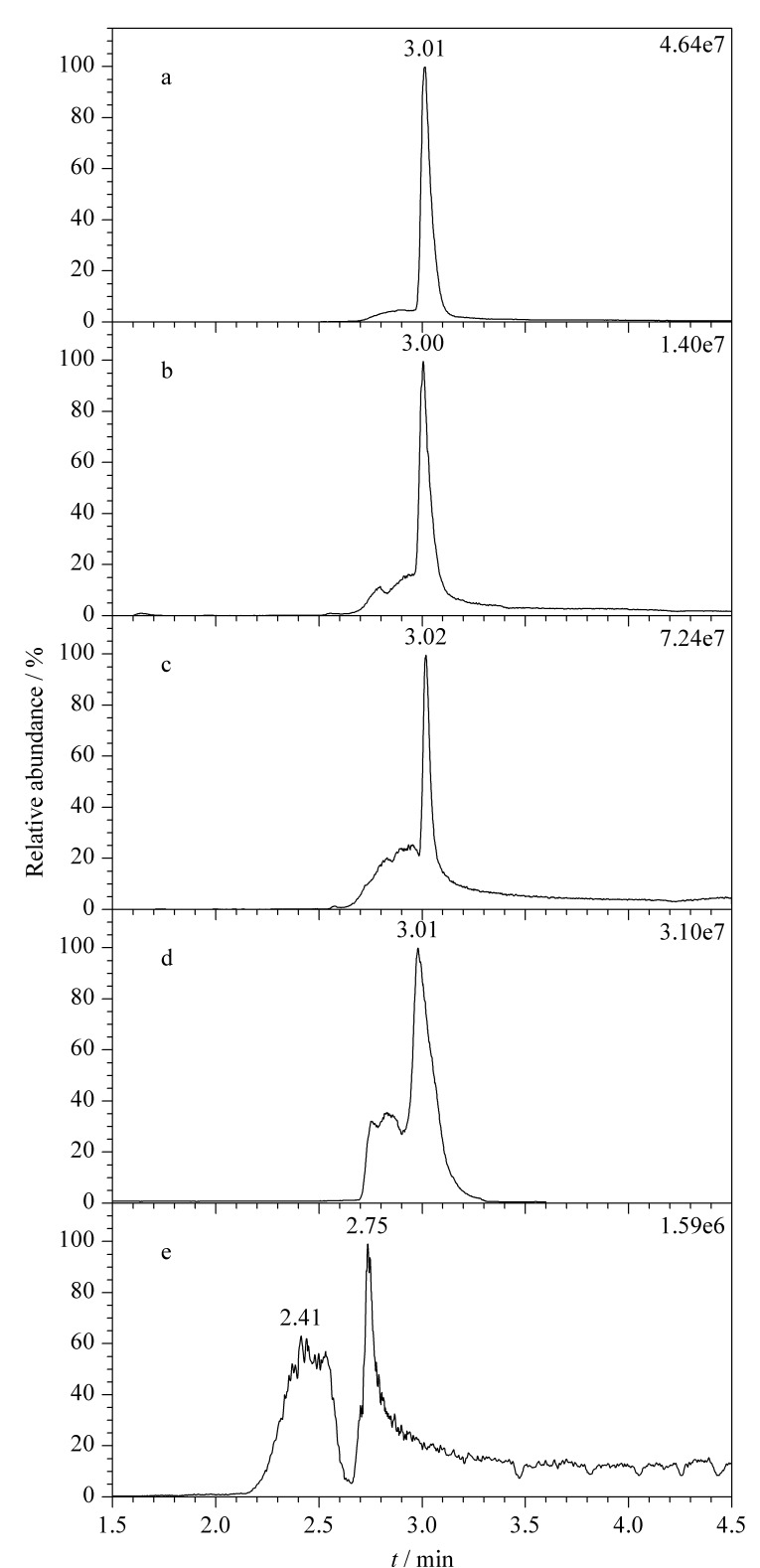
采用不同提取剂时腺苷的MRM色谱图

### 2.2 方法学验证

#### 2.2.1 基质效应

通过鼠心脏样品中腺苷的信号抑制/增强程度(signal suppression/enhancement, SSE)来评估基质效应^[23]^。SSE=*S*_1_*/S*_2_×100%,其中*S*_1_和*S*_2_分别为基质标准曲线的斜率和空白溶剂标准曲线的斜率。当SSE值大于100%,表明存在基质增强效应;SSE值低于100%,表明存在基质抑制效应;SSE值为80%~120%时,表明基质效应影响较小。实验结果表明,基质标准曲线线性方程为*Y*=52290.1*X*+84918.6,相关系数(*r*^2^)=0.9930,溶剂标准曲线线性方程为*Y*=59221.9*X*+70115.2,*r*^2^=0.9930。其中*X*为腺苷的质量浓度(ng/mL),*Y*为信号响应强度。SSE值为113.26%,在80%~120%范围内,说明鼠心脏组织中腺苷的基质效应较小,可以忽略,不需要补偿基质效应,因此本实验采用溶剂标准曲线对心脏组织样品进行定量分析。

#### 2.2.2 线性关系、LOD和LOQ

利用超纯水对腺苷标准储备液进行稀释,得到不同质量浓度的系列溶剂标准工作溶液,以腺苷的质量浓度(*X*, ng/mL)为横坐标,信号响应强度为纵坐标(*Y*),建立腺苷的溶剂标准曲线;腺苷的LOD和LOQ分别以3倍和10倍信噪比确定。结果表明,腺苷在0.1~160 ng/mL范围内线性关系拟合较优,LOD和LOQ分别为0.03 ng/mL和0.1 ng/mL。

#### 2.2.3 回收率和精密度

按照1.2节样品前处理条件进行加标回收和精密度试验,在空白鼠心脏样品基质中分别添加低、中、高(0.1、10、80 ng/mL, *n*=6)3个不同水平的腺苷标准工作液,根据测定值和理论值的百分比计算各自的回收率,日内精密度和日间精密度分别为同一天6次平行试验和不同自然日(*n*=3)测定结果的相对标准偏差。结果表明,腺苷在鼠心脏组织中低、中、高3个不同水平下的平均回收率分别为113.6%、96.3%、102.9%,日内精密度分别为8.4%、3.5%、1.7%,日间精密度分别为2.6%、7.4%、5.5%。以上结果表明,所建立的心脏组织中腺苷检测方法结果准确可靠,可用于实际样品的分析。

### 2.3 实际样品检测

采用本文建立的UPLC-MS/MS法同时测定34份鼠心脏组织(17份大鼠和17份小鼠)中腺苷含量,测量结果如图3所示。在17份大鼠心脏组织中,腺苷含量范围为10.24~15.19 mg/kg,平均含量为12.60 mg/kg;在17份小鼠心脏组织中,腺苷含量范围为3.31~8.75 mg/kg,腺苷平均含量为5.37 mg/kg。

**图 3:**
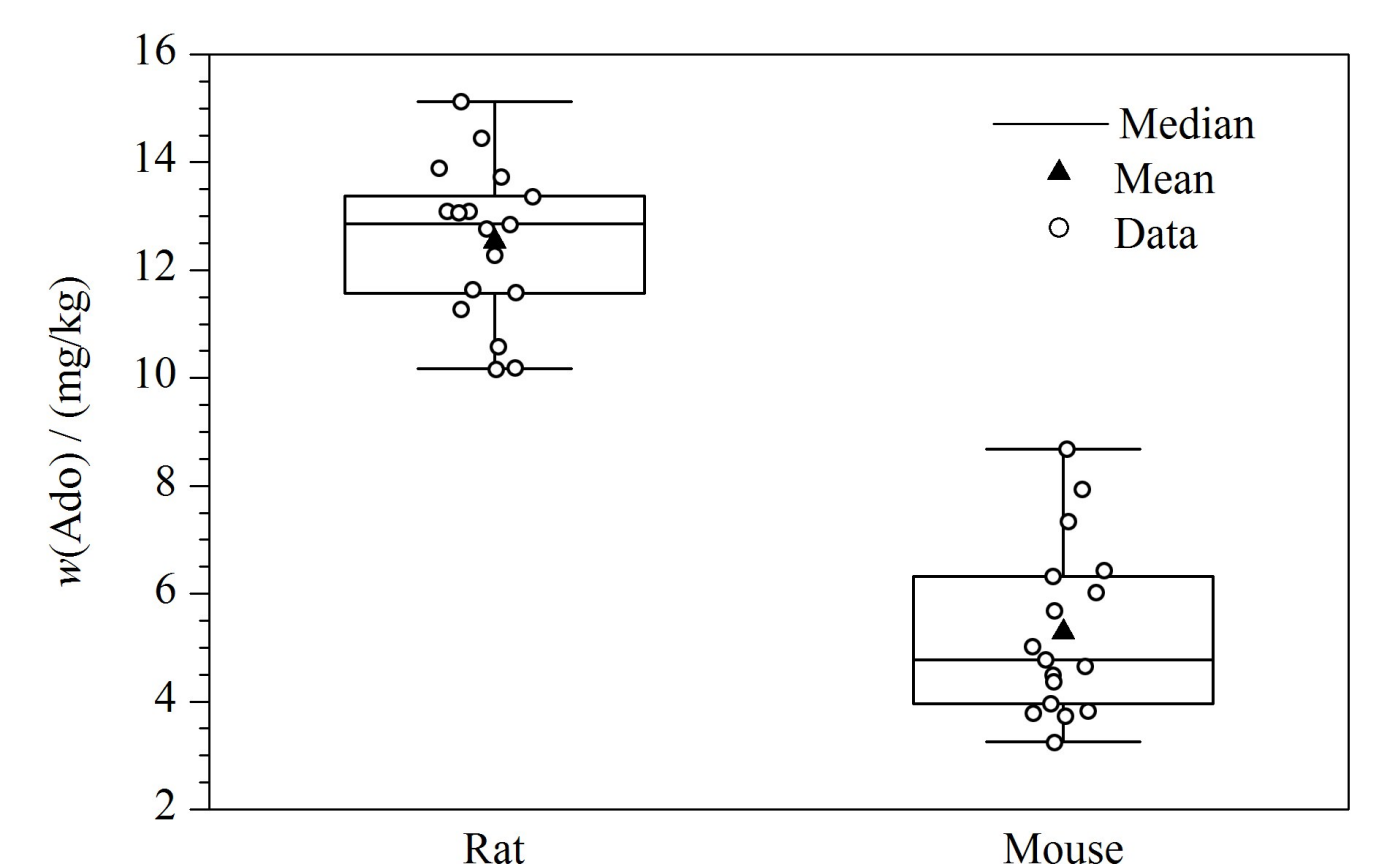
心脏组织中腺苷的含量水平

### 2.4 两种腺苷检测方法的相关性和一致性分析

采用两种检测方法同时对34份鼠心脏组织(17份大鼠和17份小鼠)中腺苷含量进行测定。如图4所示,以双抗体夹心法的测定值为横坐标(*X*, mg/kg), UPLC-MS/MS法的测定值为纵坐标(*Y*, mg/kg),拟合回归方程:*Y*=0.9894*X*-2.198, *r*^2^=0.9226, *P*<0.0001,拟合方程具有统计学意义,两个方法的检测结果均匀分布于回归方程两侧,表明两个检测方法的腺苷检测结果具有极强的线性相关关系。

**图 4:**
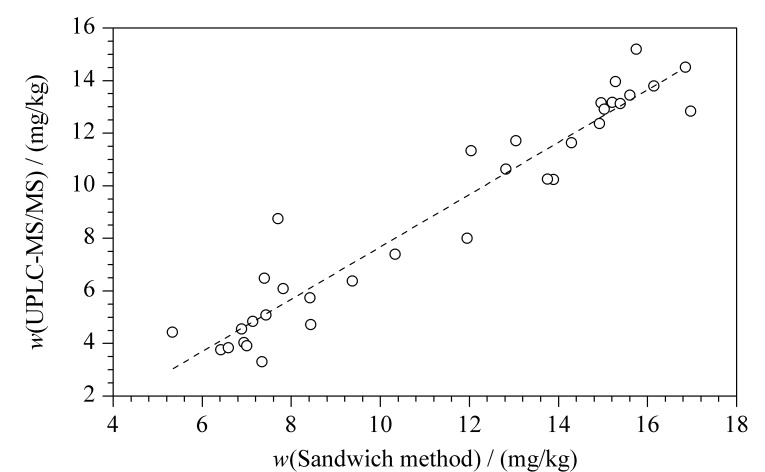
两种腺苷测定方法的相关性检验

应用GraphPad Prism 9.0.0软件中的Bland-Altman散点图,分析UPLC-MS/MS法和双抗体夹心法两种方法测量结果的一致性,如图5所示。Bland-Altman分析显示两种方法测量腺苷含量差值的95%一致性界限LOA为0.1531~4.4830 mg/kg,虽然97.06%的点落在95%的一致性界限范围以内,但双抗体夹心法检测值较UPLC-MS/MS法存在显著正偏倚。双抗体夹心法检测结果值高于UPLC-MS/MS法的测定值,差值的绝对值最大为4.130 mg/kg,差值的平均值为2.318 mg/kg,差值的标准差为1.104。综上,两种方法在心脏组织中腺苷的检测中存在一定程度的偏差,这可能是由于所选用的双抗体夹心法主要是用于小鼠血清中腺苷含量的测定,心脏组织由于基质复杂,在腺苷含量检测上有一定的偏差,不适用于心脏组织中腺苷的测定。目前国内外关于鼠心脏组织中腺苷含量的测定方法鲜有报道,本文所建立的方法可以填补该领域的空缺。

**图 5:**
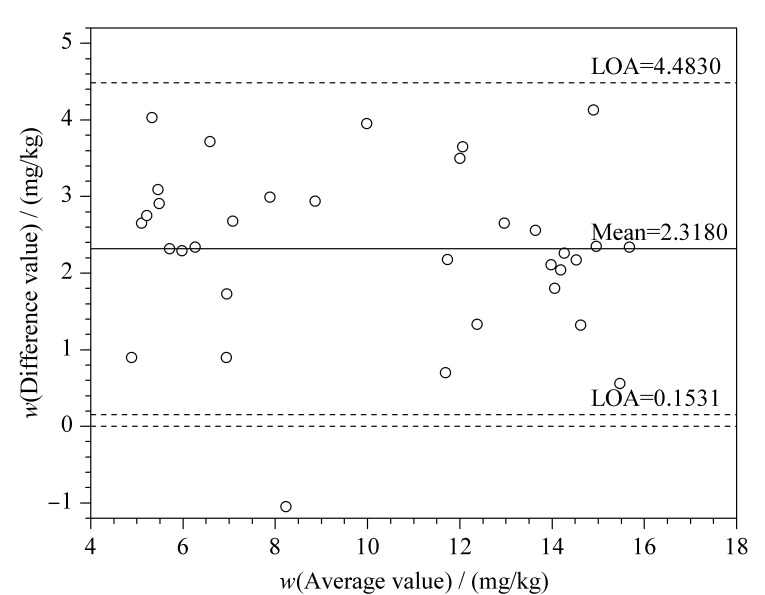
两种腺苷测定方法的一致性检验

### 2.5 方法比较

通过与目前已经报道的文献对比(见表2),采用UPLC-MS/MS测定鼠心脏组织中的腺苷含量,由于基质不同,各方法的灵敏度有一定的差别,但总体上,本文所建立的方法更灵敏,操作更简单。

**表 2:** 本文所建立的方法与文献报道的检测方法对比

Method	Samples	LOD	Ref.
Electrochemical method	plasma and serum	1.0 mmol/L (0.27 ng/mL)	[14]
Electrochemical method	plasma	8.3 mmol/L (2.22 ng/mL)	[15]
Electrochemical method	in vivo	0.1 mmol/L (0.026 ng/mL)	[16]
Hydrogel aptasensor	serum and urine	0.09 mmol/L (0.024 ng/mL)	[17]
LC-UV	health foods	1000 ng/mL	[18]
UPLC-MS/MS	cordyceps products	0.01 ng/mL	[19]
HILIC-MS/MS	serum and urine	0.01 mmol/L (0.026 ng/mL)	[24]
UPLC-MS/MS	heart tissue	0.03 ng/mL	this work

HILIC: hydrophilic interaction liquid chromatography.

## 3 结论

本研究建立了基于UPLC-MS/MS快速高效检测心脏组织中腺苷含量的方法,在提取剂中加入EHNA稳定剂预防腺苷代谢及其在提取过程中的降解,通过对色谱、质谱、提取剂等条件进行优化,有效减少了基质干扰,提高了心脏组织中腺苷的检测灵敏度。与传统腺苷检测方法相比,本方法检测灵敏度高,特异性好,操作简单,且能够快速准确检测组织样品中腺苷含量,可为临床研究和疾病诊断提供技术支撑。
